# Fully Automated Microsystem for Unmediated Electrochemical Characterization, Visualization and Monitoring of Bacteria on Solid Media; *E. coli* K-12: A Case Study

**DOI:** 10.3390/bios9040131

**Published:** 2019-11-04

**Authors:** Cesar A. Hernandez, Valerio Beni, Johann F. Osma

**Affiliations:** 1 CMUA. Department of Electrical and Electronic Engineering, Universidad de los Andes, Carrera 1E # 19A-40, Bogota 111711, Colombia; ca.hernandez11@uniandes.edu.co; 2 Biosensors and Bioelectronics Centre, Department of Physics, Chemistry and Biology (IFM), Linköping University, S-58183 Linköping, Sweden; valerio.beni@ri.se; 3 Department of Printed Electronics, RISE Acreo, Research Institute of Sweden, 16440 Norrköping, Sweden

**Keywords:** bio-electrochemical systems, microsystem, bacteria, direct electron transfer, cyclic voltammetry, electrochemical impedance spectrometry, microscopy, automated system

## Abstract

In this paper, we present a non-fluidic microsystem for the simultaneous visualization and electrochemical evaluation of confined, growing bacteria on solid media. Using a completely automated platform, real-time monitoring of bacterial and image-based computer characterization of growth were performed. Electrochemical tests, using *Escherichia coli* K-12 as the model microorganism, revealed the development of a faradaic process at the bacteria–microelectrode interface inside the microsystem, as implied by cyclic voltammetry and electrochemical impedance spectrometry measurements. The electrochemical information was used to determine the moment in which bacteria colonized the electrode-enabled area of the microsystem. This microsystem shows potential advantages for long-term electrochemical monitoring of the extracellular environment of cell culture and has been designed using readily available technologies that can be easily integrated in routine protocols. Complementarily, these methods can help elucidate fundamental questions of the electron transfer of bacterial cultures and are potentially feasible to be integrated into current characterization techniques.

## 1. Introduction

Understanding the behavior of live bacterial cells is a subject of continuous interest, with implications for the food industry, environmental studies, medicine, agriculture, water treatment, energy production, chemical synthesis, biosensors, and other industrial and biotechnological applications [1]. A reason for this interest is the ability of bacteria to ubiquitously adapt to different environments [2]. This ability is regarded as the result of evolutionary changes induced in bacterial regulation strategies as a response to energetic requirements related to the respiratory system [3,4].

The bacterial respiration process is a collection of electrochemically governed mechanisms that allow electron transport to derive the required energy for cell survival [5]; some bacteria can exchange electrons with a broad range of external acceptors/donors [6,7] through membrane associated complexes [8,9]. Albeit all microorganisms can be considered electroactive in the respiration process [10], only 94 species, from bacteria and archaea, have been formally characterized as such [11]. In addition, some bacteria can readily exchange electrons with solid substrates, e.g., metals and minerals, through a variety of mechanisms [12]; these processes are usually referred to as external electron transfer (EET), and are particularly attractive for research [12,13,14,15], as they can be integrated into electric and electrochemical systems, e.g., via conductive electrodes. The interactions performed at the electrode–bacteria interface can be of an anodic (substrate oxidation) [16], or cathodic (substrate reduction) [17] nature, and the related redox response acts as a sensory system that connects the internal mechanisms of the bacteria with the environment; in this way, bacteria regulate the production of organic compounds and gene expression [18].

In this context, the use of bacterial electrochemical properties has been proposed for the development of bio-electrochemical systems (BES) [19,20,21,22,23]; however, despite the efforts invested in research, BES have not reached the technological maturity to permeate commercial applications [24,25]; moreover, the mechanisms involved with the bacterial EET remain largely unknown [11,13,20,23,26,27,28]. Consequently, the need to revisit fundamental research on the EET mechanism has been identified [13,14,20,29,30]. Current work must be directed to the (r)evolution of standardized systems, terminology and techniques to characterize BES [28,31,32,33]; in this sense, constructing a solid framework to better describe, compare and improve the performance of upcoming BES advances and applications.

Currently, bacterial BES are largely reliant on bacterial suspension cultures of isolated or multi-species consortia; bacteria are either expected to form biofilm structures at the electrode surface or to produce mediated reactions with the electrodes. In the case of biofilm formation, multiple factors (both intrinsic and extrinsic) can influence their structure and adherence [34,35], increasing the complexity of the EET mechanism and thus the analysis of the involved phenomenon. For instance, Xie et al. present an electrochemical quartz crystal microbalance (EQCM) to study adaptation of *E. coli* to different redox controlled environments [36]. In this microsystem, the formation of a biofilm on a gold-coated quartz electrode was advantageous for bacterial proliferation; furthermore, electroactivity of the bacteria was reported after 18 h of exposure to the redox controlled environment. Besides this specific application, there are different parameters that must be studied in order to work with electroactive biofilms, such those stated by Babauta et al. [10], including techniques, system configuration, and modelling, among others.

Microsystems have been successfully used as an alternative for the study of complex redox processes that take place within bacterial cells and their immediate environment [37,38,39]. The use of microfluidic systems provides tools to examine cells, from the individual to the population level [40], and their extracellular surroundings with precise control of the environment and physiological conditions [41,42,43,44,45]. In this scope, the work of Fraiwan is interesting, as he presents a bio-microsystem to simultaneously observe and perform electrochemical evaluation of microbial cells [46].

Notwithstanding several advantages in the use of microfluidics for the study of the bio-electrochemical interactions of bacteria, the effect of shear stress, which influences the formation and maintenance of bacterial biofilm structures [47], induces further complexity to the control and modelling of these systems; moreover, microfluidics poses the challenge of sustainability for long-term live-cell imaging, and functionality towards effective biomolecule detection [40].

An alternative microsystem has been proposed for the culture of bacteria in a confined environment on a solid substrate [48,49], where the growth characteristics and a monitoring system for *Escherichia coli* MG1655 were previously discussed [49]. Bacteria under this type of confined growth has been recognized to transit between a two-dimensional (single layer) to three-dimensional growth [49,50]. This characteristic is related to the “expansion growth” generated by the pushing of the new generation of cells towards each other [51].

We present a non-fluidic microsystem that takes advantage of this property, using an optically transparent confinement microstructure, bacteria inside the microsystem is forced to grow in close contact with a conductive surface (microelectrodes). The microsystem integrates temperature and monitoring control systems to traditional optical techniques and electrochemical techniques, which grants autonomy for long term sustainability. Furthermore, the microsystem has been designed to the best extent using fast prototyping tools, and, when not possible, technologies that are commonly integrated into microfabrication research facilities or are commercially available. We expect that the discussed microsystem could be adopted for the routine characterization of bacterial BES and would significantly contribute to the exploration of the mechanisms of EET.

## 2. Materials and Methods

### 2.1. Reagents and Equipment

All solutions were prepared using Milli-Q water (Millipore Merck KGaA, Darmstadt, Germany). Bacterial culture was performed using bacteriological agar and LB broth (Miller) purchased from Scharlau (Scharlab, S.L., Barcelona, Spain). LB liquid medium was prepared at 25 g/L LB broth content, while LB agar was prepared at 25 g/L LB broth and 15 g/L bacteriological agar content. Spectrophotometric measurements for optical density at a wavelength of 600 nm (OD_600_) were performed using a Genesys 20 Visible Spectrophotometer (Thermo Fisher Scientific, Waltham, MA, USA).

For the fabrication of microsystems and copper-based glass heaters, borosilicate glass slides of 25.4 mm × 76.2 mm × 1.2 mm were purchased from vendor Sail Brand (Yancheng, China). Parafilm M^®^ was purchased from Bemis Company (Oshkosh, WI, USA). Design of photolithographic masks was made using EAGLE 8.3.2 PCB Design Software (Autodesk, San Rafael, CA, USA). Positive photo resist MICROPOSIT™ SC™ 1827 (SC-1827 photo resist) and developer MICROPOSIT™ MF™ 319 were purchased from Rohm and Haas Electronic Materials LLC (Marlborough, MA, USA). Negative photoresist HARE SQ-25 (SQ-25 photo resist) and HARE Developer were purchased from KemLab (Woburn, MA, USA). Baker PRS-1000 stripper for lift-off was purchased from Avantor (Radnor, PA, USA). Gold at 99.99%, chromium at 99.95% and copper at 99.99% were purchased from Kurt J. Lesker Company (Clairton, PA, USA). Potassium hydroxide (KOH) was acquired from E K Industries, Inc. (Joliet, IL, USA). Sonication was carried out using a Branson CPX2800H sonicator (Branson Ultrasonics Corp., Danbury, CT, USA). Spin coating was performed using a SPIN150 spin coater (SPS Europe B.V., Putten, The Netherlands). UV exposure for photolithographic processes was performed using a Karl-Suss MJB-3 Aligner (SÜSS MicroTec SE, Garching, Germany). Metal physical vapor deposition (PVD) was achieved using an Edwards E306 evaporator (Moorfield Nanotechnology Limited, Knutsford, Cheshire, UK). Profilometry was carried out using a Dektak 3 profilometer (Veeco, Plainview, NY, USA). Resistivity and conductivity testing were performed using a digital multimeter Fluke 79 III (Fluke, Everett, WA, USA).

Microelectrode cleaning and testing were carried out using isopropyl alcohol, sulfuric acid and potassium chloride (KCl) of analytic grade. Potassium ferricyanide (III) and potassium hexacyanoferrate (II) trihydrate were purchased from Sigma-Aldrich (St. Louis, MO, USA). Ferro/ferri solution was prepared at a concentration of 2.5 mM potassium ferricyanide (III) and 2.5 mM potassium hexacyanoferrate (II); a concentration of 0.1 M of KCl was added to the solution. Electrochemical procedures were performed using a PalmSens3 EIS Potentiostat with proprietary software PSTrace (PalmSens BV, Houten, The Netherlands). The impedance response of the microsystem was analyzed using an EIS Spectrum Analyzer 1.0 (Alexander S. Bondarenko and Genady A. Ragoisha).

Three-dimensional printed elements were fabricated using a Monoprice MP Select Mini 3D Printer V2 with polylactic acid (PLA) filament Monoprice MP Select PLA Plus (Monoprice Inc., Rancho Cucamonga, CA, USA). Three-dimensional models were created using Solid Edge ST (Siemens, Munich, Germany). Optical observation was performed using an Olympus CX21 microscope (Olympus, Shinjuku, Tokyo, Japan), coupled with a Sony DSC-QX10 camera (Sony, Minato, Tokyo, Japan). For the stage movement of the automated monitoring microsystem, two Astrosyn SST-024 stepper motors (Astrosyn International Technology Ltd., Chatham, Kent, UK) and one Futaba S33 servomotor (Hobbico Inc., Champain, IL, USA) were used. In-house software for hardware control, data acquisition and data analysis were developed in Microsoft Visual Studio (Microsoft, Redmond, WA, USA) and MATLAB R2016a (MathWorks, Natick, MA, USA).

### 2.2. Microorganism

*Escherichia coli* K-12 strain MG 1655 [52] was obtained from the biophysics laboratory’s cell culture collection at Universidad de Los Andes (Bogota, Colombia). *E. coli* was used as a model organism in light of a well-known respiratory system, with an accepted model for the investigation of its energetics [9,36,53,54,55,56,57].

Bacteria was recovered weekly from −80 °C storage, grown on LB agar for 18 h, and stored at −4 °C. Single colony harvesting was performed from individual petri dishes.

### 2.3. Design of the Microsystem: Microelectrodes and Confinement Microstructure

**Figure 1:**
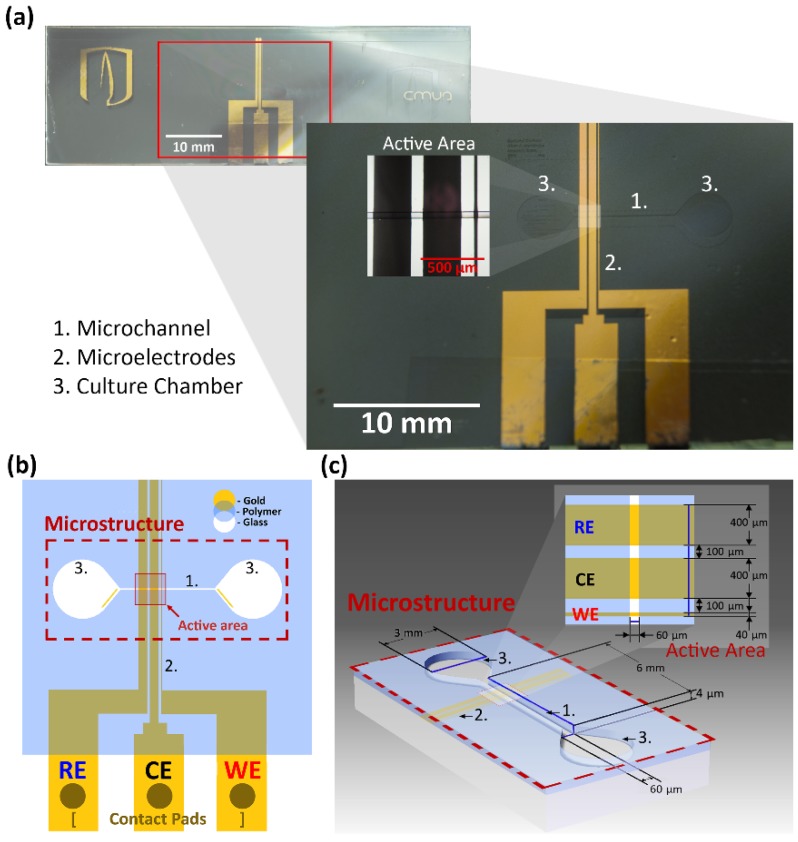
(**a**) Photograph of the built microsystem. Inset: active area, detailed micrograph of the confinement structure and microelectrodes. (**b**) Layout diagram of the confinement microstructure and microelectrodes (microstructure and active area indicated). (**c**) Dimensions diagram of the confinement microstructure (ss shown in (**b**)). Inset: active area dimensions diagram. RE = reference electrode; CE = counter electrode; WE = working electrode.

Microsystems for bacterial confinement and electrochemical measurements were built on borosilicate glass slides (Figure 1a); each microsystem was provided with a confinement microstructure (Figure 1b), built by means of a thin polymeric SQ-25 photo resist layer (see Section 2.3.2), placed ~5 mm from the edge of the contact pads, with three embedded gold microelectrodes (Figure 1b) fabricated by means of a deposited thin gold layer (see Section 2.3.1). All electrochemical measurements were performed at the microstructure–microelectrode intersection, referred to in this work as the active area (Figure 1a–c). This configuration constrained the bacterial growth towards, allowing the bacteria to be in close contact with the microelectrode’s surface.

#### 2.3.1. Microelectrodes

Microelectrodes (Figure 1b,c) were fabricated using a photolithographic lift-off technique. The working electrode (WE), with an active surface area of 40 µm × 60 µm, counter electrode (CE) and reference electrode (RE), both with an active surface area of 400 µm × 60 µm, were distinctively endowed with 3 mm × 6 mm contact pads separated 2 mm from each other (Figure 1b).

Borosilicate glass slides were immersed in a 1 M KOH solution and sonicated for 10 min, rinsed with Milli-Q water, air dried, and heated on a hotplate at 120 °C for 5 min. The glass slides were spin-coated with SC-1827 photo resist at 5000 RPM for 1 min and pre-cured at 100 °C for 50 s on a hotplate. Coated glass slides were exposed to UV light using a photolithographic mask, developed and exposed directly to UV light for 1 min. Deposition of a 20 nm chrome adhesion layer and subsequently an 80 nm gold layer was performed by means of PVD. The glass slides were submerged in stripper to remove the remaining photo resist, and submerged in a 1 M sulfuric acid solution for 1 min, rinsed with Milli-Q water, air dried and heated on a hotplate for 5 min at 120 °C.

#### 2.3.2. Confinement Microstructures

Confinement microstructures were built on top of the glass slide’s microelectrode surface (Figure 1b). Selected glass slides were submerged in a 1 M sulfuric acid solution for 1 min, rinsed with Milli-Q water, air dried and heated on a hotplate for 5 min at 120 °C. They were then spin-coated with SQ-25 photo resist at 5000 RPM for 60 s, soft baked on a hotplate for 50 s at 65 °C and exposed with a photolithographic mask to UV light. A post-exposure bake was performed on a hotplate at 95 °C for 60 s; then, the photo resist was developed followed by a final exposure to UV light for 120 s and heating at 120 °C for 5 min. Each confinement microsystem contained a 3 mm culture chamber connected through a 6 mm × 60 µm × 4 µm microchannel (Figure 1c).

### 2.4. Test Cell Design

A test cell was built to contain the bacterial microculture and to isolate the microsystem from the environment. The proposed design allows direct real-time observation, facilitates temperature control and enables connections from the microsystem to external devices.

**Figure 2:**
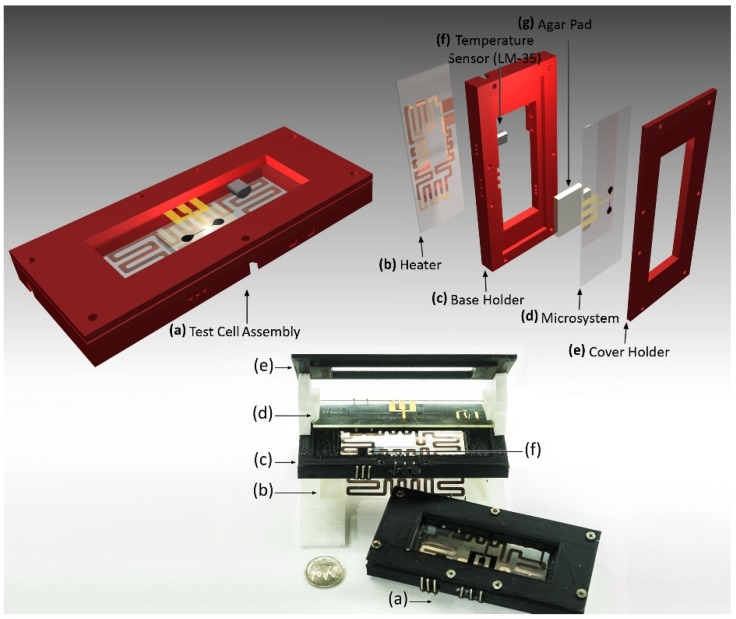
Test cell three-dimensional (3D) representation and photograph. (**a**) Assembly. (**b**) Copper-based glass heater. (**c**) PLA base holder. (**d**) Microsystem (microelectrodes and confinement microsystem). (**e**) PLA cover holder. (**f**) Temperature sensor (LM-35). (**g**) Agar pad.

The assembled test cell (Figure 2a), with a total volume of 9 cm × 4 cm × 1.5 cm, contained a transparent copper-based glass heater (Figure 2b) joint and was sealed with silicone to a 3D-printed PLA base holder (Figure 2c), a LM35 temperature sensor (Figure 2f) and a 3D-printed PLA cover holder (Figure 2e). To conduct the experiments, an agar pad (Figure 2g) was used to contain the bacterial cells inside the microsystem (Figure 2d), whilst providing them with a nutritive medium. The microsystem with the adhered agar pad was placed between the base holder and the cover holder and secured using seven 2 mm screws.

The copper-based glass heater, with a 100 nm copper layer and a mean resistance of 60 Ω as measured between contact pads, was fabricated using the photolithographic lift-off process (similar to those previously described for the fabrication of the microelectrodes). The copper heating resistance was protected using a generic transparent varnish. The closed-loop internal temperature control of the test cell was performed using an automated monitoring system.

### 2.5. Automated Monitoring System

Complementary hardware and software systems were developed to acquire and manage images and information of and from the test cell experimental setup [49]. A set of 3D-printed components were designed and coupled to the microscope. Computer-controlled movement of the microscope X–Y stage (76 mm × 30 mm), adjusted for a 15 µm step advance, was achieved from the adaptation of two stepper-motors to each of the microscope stage knob shafts using a 1:5 pulley-belt transmission. Fine focus Z axis stage movement, for a range of 150 µm, was actuated with the direct coupling of a 180° servomotor to the fine focus knob. A digital camera was coupled to the microscope eyepiece tube. An image resolution of 4 pixel/µm was achieved for a 10× microscope objective, 10× camera optical zoom and 2 M (1920 × 1080) image resolution camera setting. Images of preprogrammed positions on the microstructure were recorded at 10-min intervals at an approximate distance of 350 µm between each.

The information of the platform position and temperature of the test cell were controlled using an Atmel ATmega2560 microcontroller and transferred via USB (Universal Serial Bus) to a personal computer (PC). An in-house program (Microscope Control, Universidad de Los Andes), received and processed the data for real-time monitoring and control of position, temperature and image acquisition events, which were stored in an automatically generated report file.

### 2.6. Bacterial Microculture

Before each experiment, in order to avoid accumulation of particles inside the microstructure, every microsystem was cleaned using isopropyl alcohol, rinsed with Milli-Q water, air dried, and heated for 5 min at 120 °C; optical inspection of the confinement microstructure was performed using the automated monitoring system to evaluate the physical integrity of the microstructures and to confirm that no physical obstruction was present inside the microchannel. All the elements of the test cell assembly, including the agar pad, were disinfected by means of UV-C direct light exposure for 30 min in a laminar flow cabinet.

Bacterial microculture was performed from a single colony harvested from storage using a sterile pipette tip. The bacterial colony was transferred into a 15 mL Falcon tube with 5 mL sterile LB liquid medium and then incubated on a 12-h overnight culture (ON) at 37 °C, 300 RPM in an orbital shaker. Following this incubation, 200 µL of the suspension was poured into a 15 mL Falcon tube with 5 mL sterile LB liquid medium and incubated at 37 °C, 300 RPM until reaching steady state at an OD_600_ between 0.3 and 0.5 [58], corresponding to a bacterial density between 1.9 × 10^8^ and 6.2 × 10^8^ colony forming units (CFU)/mL. Suspended cells were diluted 1:10 in sterile LB liquid medium to be used as inoculum.

**Figure 3:**
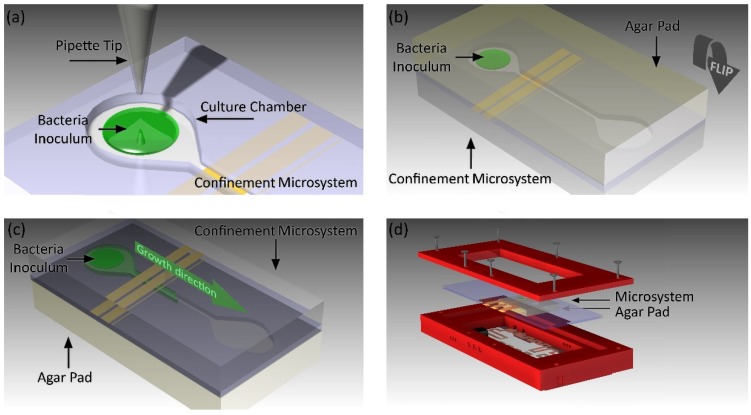
Diagram of the microculture process. (**a**) Inoculum (1 µL) was placed inside one of the culture chambers. (**b**) Carefully, an agar pad is placed on top of the microsystem. (**c**) The microsystem was turned over, allowing bacterial growth towards the microelectrodes. (**d**) The microsystem was placed inside the test cell.

Using a micropipette, 1 µL of inoculum (~4.05 × 10^4^ CFU) was deposited inside one of the culture chambers in the microsystem (Figure 3a) and left to dry for 4 min at room temperature to obtain a bacterial smear of ~2 mm diameter; in this way, overflooding of the inoculum in the culture chamber was avoided, as the initial volume of the drop was reduced due to evaporation of the liquid contents. Once the liquid of the inoculum was completely evaporated, an LB agar pad (20 mm × 15 mm × 3 mm) was placed on top of the confinement microsystem to seal the bacteria within the culture chamber and the LB agar pad (Figure 3b). The agar pad was left to adhere for 30 s to the microsystem and then faced down (Figure 3c). The microsystem was positioned inside the test cell and secured using screws (Figure 3d). Parafilm strips were placed between the cover and the base holder to reduce evaporation of the test cell contents and the entry of undesirable particles and microorganisms. The test cell was placed into the automated monitoring system and the temperature was maintained at 37 °C during experimentation.

All microsystems were optically inspected before and during the length of the experiment. Microsystems were rejected if there were non-identified particles inside the microstructure before initiating measurements, if there were bubbles at the microelectrode surface or bubble formation that might interfere with the bacterial growth.

In order to verify the sterilization process, control experiments were performed using the previously described processes without inoculation of the microsystem. Microsystems were monitored for over 24 h; images of different segments of the microsystems were captured at 10-min intervals using the automated monitoring system (see Section 2.4). During the monitoring, no severe drying of the agar was noticed, and no growth of any type of microorganism was detected, ensuring the aseptic conditions of the microsystem prior to inoculation (Figure A2).

### 2.7. Electrochemical Procedures

Electrochemical measurements were performed at the active area. Prior to bacterial growth, the channel was empty and did not contain culture media or any fluid; once the bacteria colonized the active area, the bacteria were in contact with the electrodes due to the “expansion growth” generated by the pushing of the new generation of cells towards each other [51]. Using a programmed computer routine, three cyclic voltammetry (CV) and one electrochemical impedance spectroscopy (EIS) cycles were executed at 30-min intervals throughout the complete growth cycle of the bacteria in the microsystem.

#### 2.7.1. Cyclic Voltammetry

Conditions for the CV measurements were determined before recording any data. Different CV scans were performed using microsystems with full grown bacteria on the active area of the electrodes; various potential ranges were evaluated in order to avoid damage to the cells and the microelectrodes (data not shown). A potential range from −0.5 V to 0.5 V was selected, as higher values resulted in immediate damage to the electrodes, while no effect on the cells was noticed. A scan rate of 50 mV/s was used.

#### 2.7.2. Electrochemical Impedance Spectroscopy

Conditions for the EIS measurements were established as follows: an equilibration time of 5 s, fixed direct current potential (DC potential) of 0 V, alternate current potential (AC potential) of 10 mV, a minimum frequency of 200 Hz and a maximum frequency of 50 KHz. No pretreatment and no post-measurement processes were performed.

### 2.8. Data Processing

#### 2.8.1. Image Analysis

Microchannel segment images were acquired using the automated monitoring system; between 10 to 15 micrographs were automatically captured every 10 min. Using in-house software (Image Analysis, Universidad de Los Andes), the images were managed, stored and processed, based on a previously reported algorithm [49].

For every set of images (acquired at the same location spot of the microsystem), color correction and contrast enhancement algorithms were applied. Then, a threshold algorithm, determined individually for each image set, was used to identify the main borders of the microchannel by employing the Hough Transform method [59]. The size and inclination of the captured microstructure were corrected, automatically aligned based on the weighted center point of the calculated area, and cropped accordingly. A second thresholding process, based on a percentile method [60], was carried out to perform texture segmentation to differentiate the bacteria colonized areas. White pixels for each of the processed binarized images, representing the presence of bacteria on the microchannel segment, were calculated and reported as a percentage based on the ratio of white pixel count to total image pixels for each discrete time instant. 

The information of the processed images was used to determine the time required for bacteria to reach the active area, this event, which indicated aggregation of bacteria over the complete surface of the electrodes, is referred in this report as the bacteria colonization event.

#### 2.8.2. Electrochemical Analysis

Electrochemical measurements were automatically executed every 30 min using PSTrace proprietary software, three CV and one EIS measurements were performed each time, the generated data of the electrochemical response and timestamps of every procedure was extracted and processed using in-house software (PSSession Reader, Universidad de Los Andes).

The analysis of electrochemical measurement data was performed for microsystems with bacterial presence across the complete surface of the active area. When no bacterial growth reached the active area (i.e., an initial inoculum was used, but the bacterial growth was below normal and did not reach the active area), or if any obstruction in the microsystem was observed, due to unidentified particles or bubbles, the experiment was terminated, and data was not used. Control experiments were performed in microsystems with no initial inoculum (no inoculum was added to the culture chamber at any moment and no contamination was observed), automated monitoring, conditions of test cell and electrochemical procedures were maintained unchanged. 

The identification of the colonization event was performed by evaluating the normalized variance of the CV current- and EIS impedance- response. In the absence of bacteria, the microelectrodes have no electric contact and the measured response is mainly influenced by stray potentials and capacitive effects, this correlates to a very low variance in the electrochemical response. After the colonization event, bacteria act as a conductive surface, creating an electric contact between the microelectrodes, this electric contact generates an increase in the current response and a decrement of the measured impedance. 

For every sample in the dataset of a measured response (*x*_1_, *x*_2_, …, *x*_M_) across M datapoints, the mean *µ_n_* of the *n*-th datapoint was calculated using Equation (1).

(1) $$\mu_{n}=\frac{1}{M}\overset{M}{\underset{i=1}{\sum }}x_{i}$$

The variance *σ*^2^*_n_* of the measured variable, calculated according to Equation (2), allowed us to perform a dynamic characterization of the microsystem response, as it relied on the specific mean of each datapoint, reducing the influence of small range noise and data outliers, while amplifying, through a quadratic effect, the large range changes.

(2) $$\sigma^{2}{}_{n}=\frac{1}{M-1}\overset{M}{\underset{i=1}{\sum }}{\left|x_{i}-\mu_{n}\right|}^{2}$$

Normalization of the variance *σ*^2^*_n_* was performed according to Equation (3) to ease the comparison between experiments that can be influenced by the microelectrode properties, such as the distance between the active area and contact pads, the differences in microelectrode resistivity, and the resistance between the contact pads and the external connectors.

(3) $$V_{n}=\frac{\sigma^{2}{}_{n}-\min\left(\sigma^{2}{}_{n}\right)}{\max(\sigma^{2}{}_{n})-\min\left(\sigma^{2}{}_{n}\right)}$$

A single step signal change was determined using Equation (4). The values *V_n_* of the normalized variances were divided in two sections determined by the minimum possible value of the *k* sample, such that the sum of the Δ deviation of the empirical estimate *χ* of each section represented the minimum deviation possible for both sections. The Δ function used was the standard deviation; the detailed develop of the function for each segment is as specified in Equation (5). The single-step change assumed at the *k*-th sample is calculated using Equation (6).

(4) $$\underset{k}{\arg\;\min}\left\{J\left(k\right)\right\}=\overset{k-1}{\underset{i=1}{\sum }}\Delta \left(V_{i}^{˙},\;\chi \left(\left[V_{1}\cdots V_{k-1}\right]\right)\right)+\overset{n}{\underset{j=k}{\sum }}\Delta \left(V_{j}^{˙},\;\chi \left(\left[V_{j}\cdots V_{n}\right]\right)\right)\;$$

where

(5) $$\overset{s}{\underset{i=r}{\sum }}\Delta \left(V_{i}^{˙},\;\chi \left(\left[V_{r}\cdots V_{s}\right]\right)\right)=\left(s-r+1\right)\log\overset{s}{\underset{i=r}{\sum }}\sigma^{2}\left(\left[V_{r}\cdots V_{s}\right]\right)\;=\left(s-r+1\right)\log\left(\frac{1}{r-s+1}\overset{s}{\underset{i=r}{\sum }}{\left(V_{i}-\frac{1}{r-s+1}\overset{s}{\underset{t=r}{\sum }}x_{t}\right)}^{2}\right)\;=\left(s-r+1\right)\log var\left(\left[\left(\left[V_{r}\cdots V_{s}\right]\right)\right]\right)$$

Hence

(6) $$\underset{k}{\arg\;\min}\left\{J\left(k\right)\right\}=\left(k-1\right)\log var\left(\left[\left(\left[V_{1}\cdots V_{k-1}\right]\right)\right]\right)+\left(n-k+1\right)\log var\left(\left[\left(\left[V_{k}\cdots V_{n}\right]\right)\right]\right)$$

The normalization algorithm distributes the values of the dataset over the complete range between 0 and 1, this means that datasets with small variation will be equally represented to datasets with large variation. To identify wether the changepoint *k* of the normalized variance represents a small or a large variation on the dataset, a change ratio is calculated. For a dataset with *M* datapoints, a representative datapoint with *n* samples (*x*_1_, *x*_2_, …, *x_n_*) and a changepoint *k* is selected, the change ratio *R* is defined as the ratio between the mean of the of the samples with an index higher than *k* (*x_k_*, *x*_*k*+1_, …, *x_n_*) to the mean of the samples with an index lower than *k* (*x*_1_, *x*_2_, …, *x*_*k*−1_), as represented in Equation (7).

(7) $$R=\frac{\frac{1}{n-k}\sum_{i=k}^{n}x_{i}}{\frac{1}{k-1}\sum_{j=1}^{k-1}x_{j}}$$

The normalized current responses variances of CV measurements were used to determine the related colonization event (Section 3.3.1), whereas current peaks variances at recorded potential points were calculated to evaluate redox activity (Section 3.3.2). The variances for the impedance magnitude and phase of the microsystem EIS response were processed similarly to the ones obtained by means of CV and used to identify the colonization event related to EIS (Section 3.4.1). The EIS data was analyzed and fitted to an equivalent circuit model (Section 3.4.2) to evaluate the evolution of the circuit parameters before, during, and after the colonization event. 

##### Cyclic Voltammetry Current Variance

Current variance analysis of CV measurements was established to find the occurrence time of the bacterial colonization event, determined as the single step changepoint of the *V_n_* normalized variance of the current response for individual samples along each experiment. The variance *σ*^2^ for every *n*-th sample with *M* current response values (*x*_1_, *x*_2_, …, *x_M_*) was calculated according to Equation (2). For experiments with *n* CV recorded samples, each sample had an *M* number of applied potential points and hence an equal number of current responses datapoints. Normalized values *V_n_* of *σ*^2^ for every *n* sample were calculated according to Equation (3).

The normalized variance data changepoint calculation was calculated as described in Equation (6), where *k* corresponds to the index of the recorded time instant in which the colonization event took place as registered by the CV. The colonization event was verified to be in agreement with the image anlysis using the PSSession software. The acquired samples had 201 applied potential ponts (*M*), with an equal number of current responses per sample. The number of samples *n* depended on the time length of the experiment as three CV measurements were performed every 30 min.

##### Identification of Cyclic Voltammetry Current Peaks

The identification of current peaks in the measured CVs (primary peaks) was performed using in-house software (PSSession Reader, Universidad de Los Andes). Extracted data of CV measurements was grouped accordingly to the result of the occurrence time of the colonization event calculated previously. Using CV current variance analysis, the two resulting groups, before and after the colonization event as registered by the CV, were processed in the same way. The variance of current magnitude for each of the applied potential datapoints was calculated according to Equation (2), and local maxima and minima were surveyed from the resulting data. As these primary peaks were not always found using this method, secondary peaks were defined as those detected using the local maxima applied to the ratio of the variance to its first derivative as well as the ratio of the first derivative of the variance to the second derivative of the variance data. Cluster analysis of the primary and secondary peaks after the CV colonization event were selected and evaluated to confirm if they represented redox activity.

##### Electrochemical Impedance Spectrometry Frequency and Phase Variance

The determination of the EIS bacterial colonization event was calculated as the single step changepoint of the *V_n_* normalized variance for the impedance and phase response for individual EIS samples along each experiment. The variance *σ*^2^ for every *n*-th sample with an *M* number of sampled frequencies with impedance and phase response values (*x*_1_, *x*_2_, …, *x_M_*), was calculated in accordance to the methodology presented for the determination of a CV bacterial colonization event for *n* samples with *M* frequencies. This result is compared to the colonization event as registered by the CV. A total of 25 frequencies (*M*) were measured in each EIS procedure. The number of samples (*n*) depended on the total duration of the experiment, as one EIS measurement was performed every 30 min.

##### Electrochemical Impedance Spectrometry Circuit Fitting

The elaboration of the equivalent circuit, based on the EIS information, was performed using an EIS Spectrum Analyzer 1.0 (Alexander S. Bondarenko and Genady A. Ragoisha). The values of the EIS dataset and the calculated mean for before and after the colonization event as registered by the CV were adjusted using the designed circuit model and represented against time. The mean values of the circuit elements, for both before and after the colonization event, as registered by the CV, were used as a reference to evaluate changes.

## 3. Results

### 3.1. Characterization of the Microsystems: Microelectrodes and Confinement Microstructure

Microsystems were tested and characterized through the different steps of the fabrication process. Prior to the fabrication of the confinement microstructures, the electrical resistance of the microelectrodes was measured from one end of the contact pads to the electrode end. Microsystems with a measured resistance above 50 Ω, for any microelectrode, were rejected.

**Figure 4:**
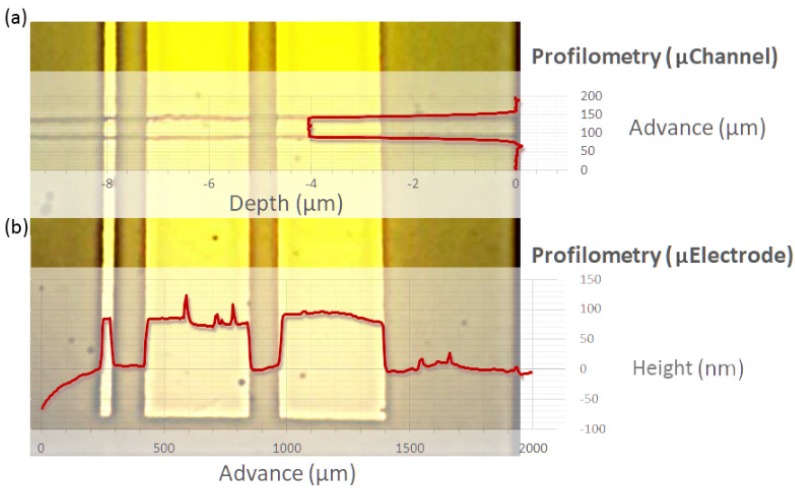
Micrograph of the microsystem active area with (**a**) representative profilometry of the microchannel structure and (**b**) representative profilometry of microelectrodes.

Profilometry was performed to determine the depth and shape of the microchannel in the microstructure (Figure 4a). The mean depth was 4 ± 1 µm, the width was 60 ± 4 µm, and perpendicularity was greater than 80°. As for the microelectrodes (Figure 4b), the height was 96 ± 14.4 nm with a distance between the microelectrodes of 100 ± 4 µm, a width of 40 ± 2.4 µm for the WE, and 400 ± 12.3 µm for the CE and RE, with a perpendicularity of 89.9°.

**Figure 5:**
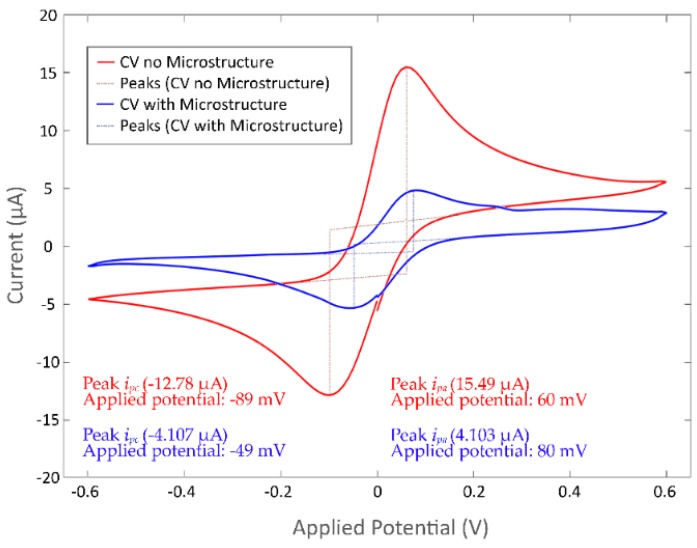
Cyclic voltammograms of ferro/ferri solution using the microelectrodes before (red) and after (blue) the fabrication of the confinement structure.

Microelectrodes were tested (three-cycle CV) before and after the fabrication of the confinement microstructure (Figure 5). CVs with potential range from −0.6 V to 0.6 V and a scan rate of 100 mV/s were recorded using a 200 µL drop of ferro/ferri solution. Before the fabrication of the confinement microstructure, ferro/ferri solution was located at a 9 mm distance from the contact pads; after the fabrication of the confinement microstructure, the ferro/ferri solution was located at the active area of the microsystem.

Current peaks of the CV mean responses were found using the value of the local maxima for the variance to a zero-value baseline. A mean cathodic current peak (*i_pc_*) of −12.78 ± 5 µA and anodic current peak (*i_pa_*) of 15.49 ± 4.5 µA were determined, with a mean potential difference between the cathodic and anodic peak currents of 149 mV for the microsystem before the fabrication of the confinement microstructure, and an *i_pc_* of −4.107 ± 1.5 µA and an *i_pa_* of 4.103 ± 1 µA, with a potential difference of 129 mV after the fabrication of the confinement microstructure. The decrease in the current values of the microsystem and the peak separation difference between the microelectrodes before and after the fabrication of the confinement microstructure were attributed to the reduction of the total area of the electrode and the difference between the distance the ferro/ferri solution was located, which would benefit the charge transfer from the electrodes to the contact pad as the distance to the microsystem is lower. Thus, the total resistance to the contact pads is also reduced.

The recorded formal potential for the ferro/ferri solution, estimated from the average of the applied potential at the anodic peak (*E_pa_*) and the applied potential at the cathodic peak (*E_pc_*), was 15.5 mV; in order to describe the results using a standard reference, the ferro/ferri formal potential is recommended to be used as internal standard [61,62], assigned to a silver/silver chloride electrode (Ag/AgCl). The defined standard redox potential of ferro/ferri vs. an Ag/AgCl electrode can be considered as 241 mV [63,64,65]. A correction of +225.5 mV is then recommended to be applied in order to compare the results to the Ag/AgCl standard potential.

### 3.2. Image Analysis

**Figure A1:**
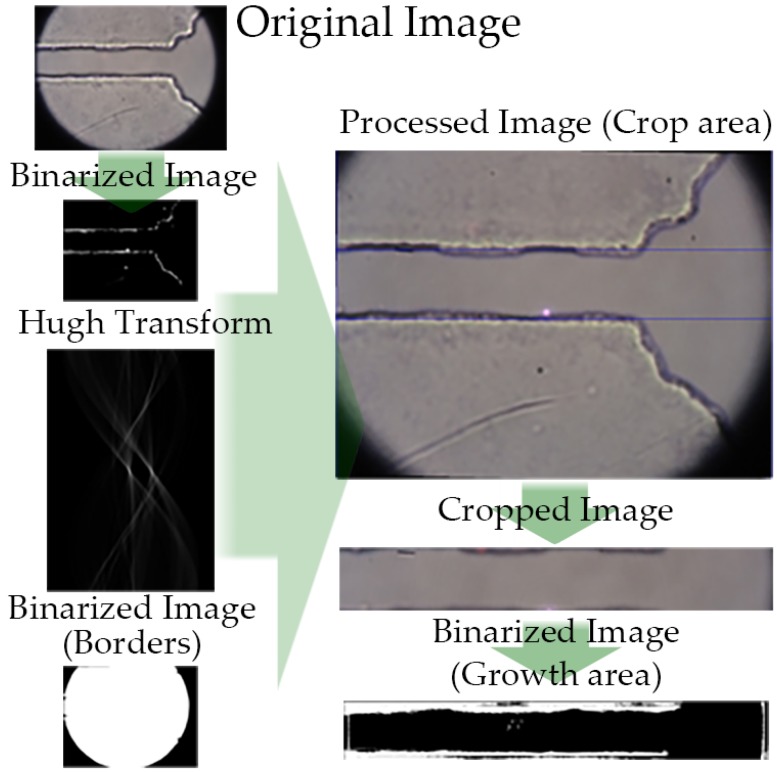
Schematic of the image analysis process. Briefly, the RAW image of the microchannel segment is binarized to detect borders. The image is then enhanced, cropped and a threshold algorithm is applied to differentiate bacterial growth from background.

**Figure A2:**
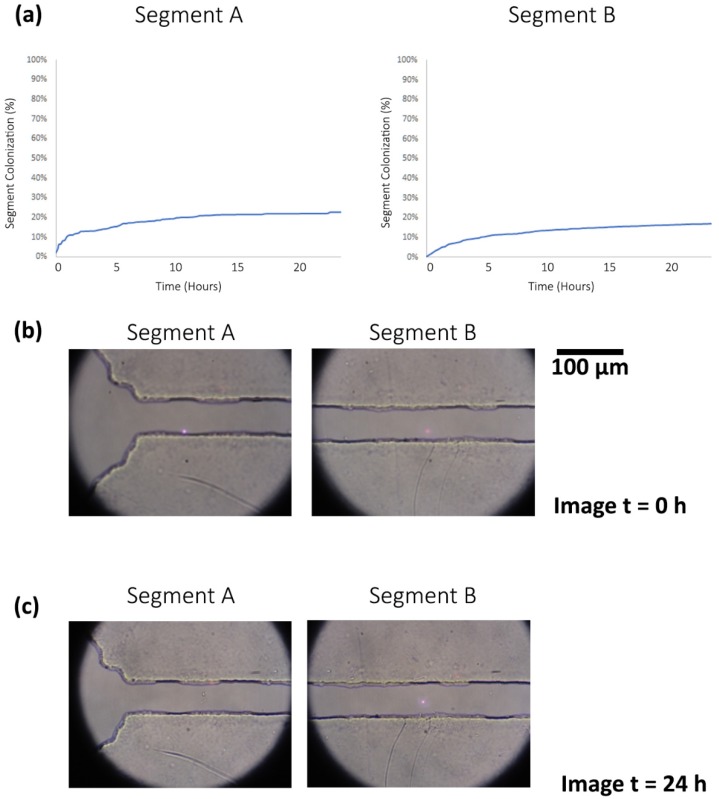
Control experiment. (**a**) Image analysis growth curve of control experiment at two different microsystem segments. RAW Images and binarized result at (**b**) t = 0 h and (**c**) t = 24 h.

Bacterial growth for each microchannel was estimated using in-house software (Image Analysis, Universidad de Los Andes) for different microchannel segments with an average area of 60 ± 4 µm × 300 ± 30 µm each (see Figure A1 for a description of the process).

**Figure 6:**
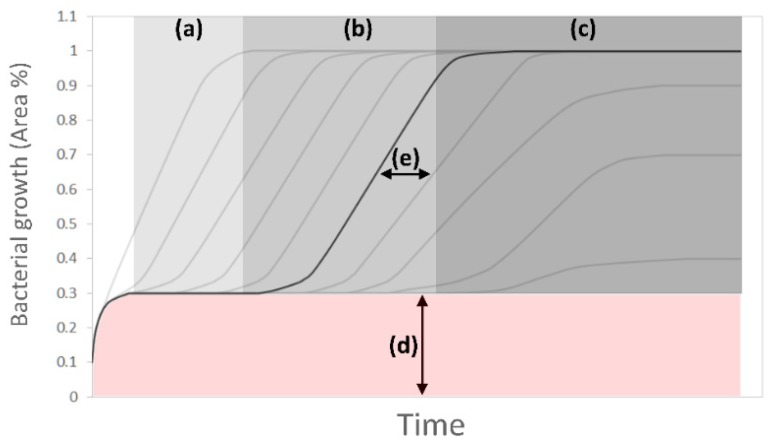
Bacterial growth model. Representation of bacterial growth calculations for different segments (gray lines). For the described segment in black: (**a**) lag phase, (**b**) exponential phase, (**c**) stationary phase, (**d**) image analysis offset, (**e**) between segment lag time.

Bacterial growth curves presented three different phases (Figure 6) similar to the phases present in traditional bacterial growth models and were named due to their similarity to them. A lag phase (Figure 6a), corresponding to the initial time in which no bacteria had reached the analyzed segment; a log or exponential phase (Figure 6b); and a stationary phase (Figure 6c), corresponding to the moment in which bacteria colonized the complete microchannel segment area, or no more growth was detected. The image analysis offset (Figure 6d) corresponded to the contribution of the microchannel borders and the cumulative image errors present in every analyzed image. The time difference, namely between segment lag time (Figure 6e), was also measured.

**Figure 7:**
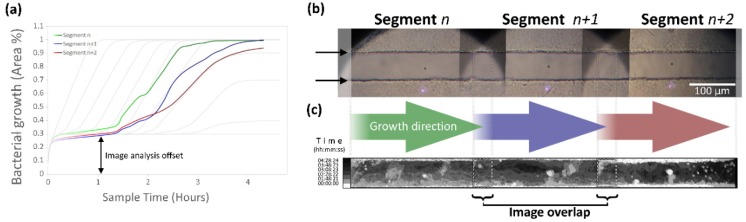
Image analysis for bacterial growth calculations (real data). (**a**) Recorded growth for the selected segments against time. The lag phase and image analysis offset can be seen. Notice that Segment *n* + 2 tends to stabilize below complete colonization of the microchannel segment. (**b**) Acquired image sample of selected segments without bacteria; the growth direction for each segment is indicated below each image. Segment *n* corresponds to a location of 1.5 mm from the culture chamber on the image. Black arrows at the left of the image indicate the microchannel edges. (**c**) Processed binarized images; the gray scale corresponds to different time points. The decrease in growth at Segment *n* + 2 and the overlapping area of the images can be seen.

The colonization of individual segments was completed in 65 ± 5 min for the selected, analyzed set of images in different experiments. The image analysis offset corresponded to 30%, and the lag phase between segments presented a variation between 30 and 60 min. For both periods, the lag phase time and the colonization time increased as the segments were further apart from the culture chamber (Figure 7a). The maximum distance reached for colonizing bacteria from the culture chambers was 5.41 mm, with a mean of 4.206 mm.

Images of segments located at the active area were not analyzed due to the light obstruction generated by the electrodes; however, it was possible to confirm growth on the microchannel by using images acquired before and after this location. Captured images were overlapped 100 µm (Figure 7b); the overlap of processed binarized images varied between 15 µm and 90 µm (Figure 7c).

The formation of colonies along the microchannel was observed to obey two different phenomena.: the expected bacterial growth of the colonies by “expansion growth” [51], and a scouting behavior [66,67]. Such behavior has been observed and described in this system before [49].

### 3.3. Cyclic Voltammetry

#### 3.3.1. Analysis of Normalized Current Variance

**Figure 8:**
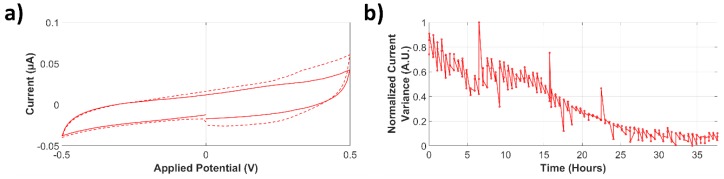
Analysis of current variance of a control experiment. (**a**) Cyclic voltammetry (CV) response of a control experiment. The dashed line represents the mean CV response during the first ~23 h. The solid line represents the mean CV current response during the following ~15 h. (**b**) Normalized current variance for a ~37-h experiment.

Occurrence time of the bacterial colonization event using CV measurement analysis was estimated using in-house software (PSSession Reader, Universidad de Los Andes). In order to evaluate if the behavior of the microsystem could be attributed to the presence of bacteria, control experiments were analyzed for over 24 h. The obtained CV response is shown in Figure 8a. A small decrease in the current magnitude can be observed between the mean values of two different segments of the dataset. Changes in the normalized current variance can be seen in Figure 8b; the graphic describes a continuous decreasing trend, which agrees to the observed CV response.

**Figure 9:**
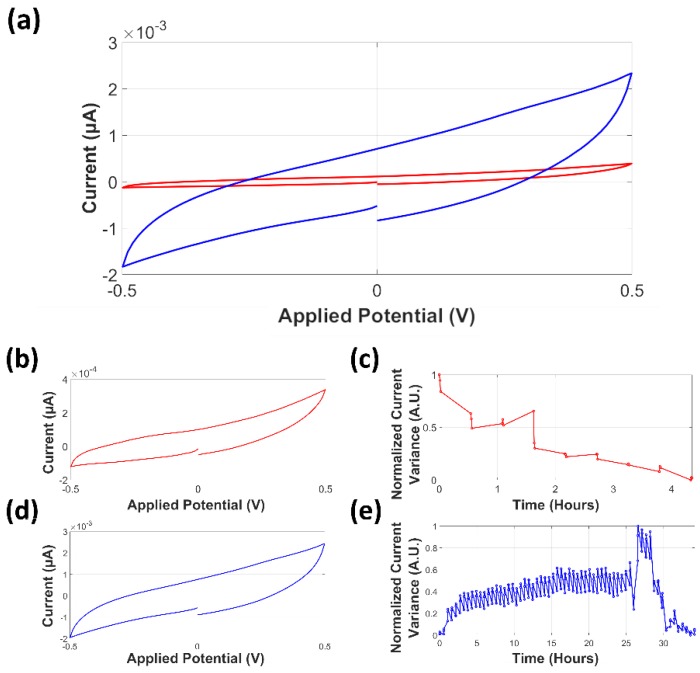
Current variance for independent sections of a colonized microsystem determined by the normalized current variance analysis. (**a**) Mean CV response for the microsystem before (red line) and after (blue line) the colonization event. (**b**) Isolated mean CV response of the microsystem before the colonization event. (**c**) Normalized current variance of the current response before the colonization event. (**d**) Isolated mean CV response of the microsystem after the colonization event. (**e**) Normalized current variance of the current response after the colonization event.

The analysis of normalized current variance for microsystems with a positive yield for bacterial growth at the full surface of the active area allowed the identification of a single step change attributed to the bacterial colonization event. This was confirmed by optical inspection and image analysis. The CV response of the colonized microsystem (Figure 9a) exhibited a notorious change in the current magnitude. The analysis of the isolated section of the CV response prior to the colonization event (Figure 9b) is shown in Figure 9c. The behavior of the microsystem during this stage is similar to that depicted by the control experiment, where no bacteria was present in the active area. The analysis of the isolated section of the CV response after the colonization event (Figure 9d) is illustrated in Figure 9e.

The images presented in Figure 9 allow us to clarify an important remark towards a correct interpretation of the results in the presented microsystem: during the development of the present project, it was observed that the current density could diverge up to three orders of magnitude between experiments. This issue was noticed for control experiments as well as for experiments with a positive yield for bacterial colonization to the active area. This difference between experiments was attributed to the influence of microelectrode properties, such as the distance between the active area and contact pads, the differences in the resistivity of the microelectrodes, and the resistance between the contact pads and the external connectors. For this reason, normalization of the variance data was applied.

The normalization algorithm that was used distributes the values of an entire dataset over the complete range between 0 and 1. This means that datasets with small variation will be equally represented to datasets with large variation, as is the case when comparing normalized variances represented in Figure 9 and is extensive to the case when comparing data of a colonized microsystem with a control experiment. The estimation of the changes in the microsystem are based on ratio measurements rather than distance measurements; as a reference, the average change ratio between current magnitude at the beginning and at the end of the experiment was ~0.5. This indicates that the current magnitude at the end of the experiment is almost half the initial measured current magnitude. Similarly, the average change ratio for the current magnitude measured at at the anodic potential of a colonized microsystem is ~8.

**Figure 10:**
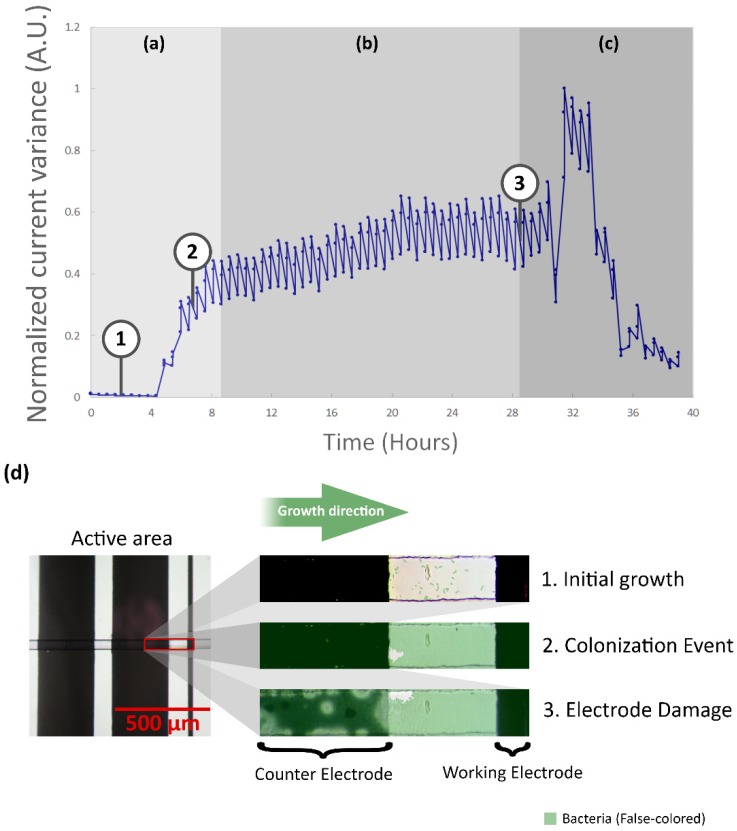
Normalized current variance analysis for a bacterial colonization event against time (real data). (**a**) Initial growth and the starting point of the bacterial colonization event stage. (**b**) Plateau stage (4 h). (**c**) Finalization of the plateau phase due to electrode damage (29 h). (**d**) Images of a microchannel segment at the active area of the microsystem during bacterial growth (false-colored) at (**1**) initial growth, (**2**) amidst the colonization event (notice the bacteria has already grown inside the microchannel segment), and (**3**) failure stage (electrode damage).

The calculated normalized current response variance for microsystems with a positive yield for bacterial growth at the full length of the active area displayed three different stages. The first is the initial growth and bacterial colonization event (Figure 10a), for which a steep increase in the normalized current response variance was observed, contrasting with the slow dynamics of the image analysis of the acquired images. The next is the colonization event, which from the perspective of the electrochemical response, happens when the bacteria is in full contact with the electrodes. This also means that it is a single curve, contrary to the various curves that compose the image analysis; the change in this curve is more abrupt; however, once the bacteria have established the electrical contact. The stabilization of the signal took from 2 h to 5 h. It was observed that the normalized variance increased correspondingly after the colonization of the segments took place, in some cases with a time delay of up to 2.5 h. The identification of the bacterial colonization event coincided with colonization observed in the image analysis. The plateau phase (Figure 10b), identified for stable behavior in the normalized current variance values with an unspecific duration for the different experiments, sustained until the failure stage, where either of two cases was observed: electrode damage (Figure 10c) or agar degradation. The microchannel segment shown in Figure 10 corresponds to a segment located at the active area. The images are labeled according to the time they were captured, as indicated at the normalized current variance. The microsystem was able to sustain the conditions for bacterial growth for over 24 h. Figure 10d shows the recorded images of a microchannel segment inside the active area in the course of the different stages. Note that the damage of the CE appeared before a sudden change in the variance of the current response that was measured.

#### 3.3.2. Current Peaks

**Figure 11:**
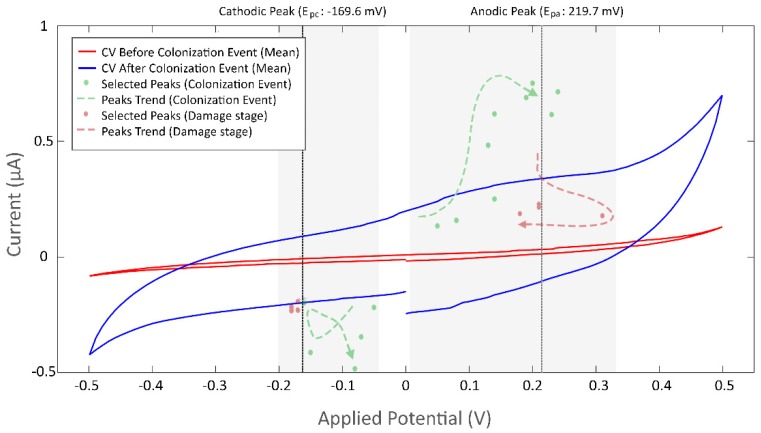
Identification of current peaks for a selected measure. Bacterial colonization was detected 4 h after initialization of the experiment and confirmed with images. Electrode damage was observed after 9 h. Mean values of a recorded cyclic voltammogram measurement before (red) and after (blue) the bacterial colonization event. Selected peaks are shown for the colonization event (green dots) and the damage stage (red dots), which was observed after 29 h. Peak trends are shown as dashed arrows for the colonization event (green arrow) and the damage stage (red arrow).

Microsystems that presented a bacterial colonization event, identified by means of image analysis and normalized current response variance, were observed to have at least one distinctive oxidation and reduction peak. The data in Figure 11 corresponds to a measurement where bacterial growth was followed by electrode damage (detailed in Figure A8).

For the anodic peak at the oxidation process during the colonization event, the scatter of selected peaks (top green dots) indicated an increasing trend for current and potential, which stabilizes at the end of the event. On the other hand, the cathodic peak at the reduction process (bottom green dots) exhibited a more hectic behavior and no trend was evidenced. During the failure stage, which occurred after 29 h of the beginning of the experiment, a strong decrease on the anodic current was observed (top red dots). It should be noted that this behavior was not constant and was regarded as tied to the cause of the decay. Cathodic peak current (bottom red dots) also presented a noticeable decrease, although with a more stable behavior. Both events can be used to identify failures in the microsystem.

Whereas current responses for the performed experiments diverged up to three orders of magnitude, the mean change ratio, measured before and after the colonization event, of the anodic current was 8.29 ± 1.26 and 6.07 ± 2.12 for the cathodic current. An anodic peak potential (E_pa_) with a mean value of 139.8 ± 27 mV and a cathodic peak potential (E_pc_) of −204.55 ± 44 mV, were identified.

### 3.4. Electrochemical Impedance Spectroscopy

#### 3.4.1. Normalized Impedance and Phase Variance

**Figure 12:**
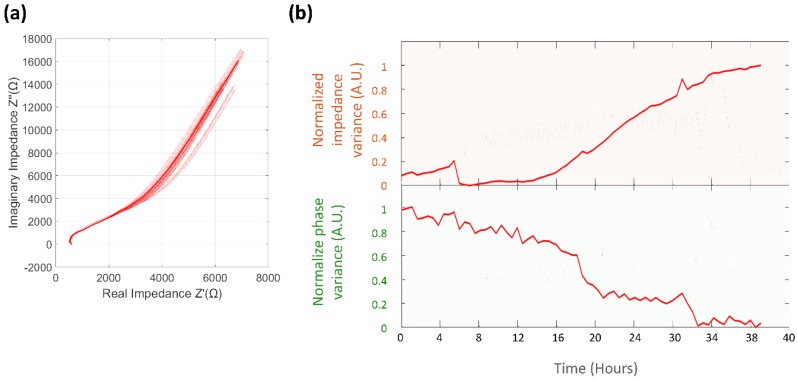
Analysis of impedance and phase variance for control experiment (**a**) EIS Nyquist plot of a control experiment, the solid line represents the mean value of EIS measurements, while the dotted lines are the actual values of the measurements during the ~37-hour control experiment. (**b**) Normalized impedance variance (Orange background) and normalized phase variance (Green background).

**Figure 13:**
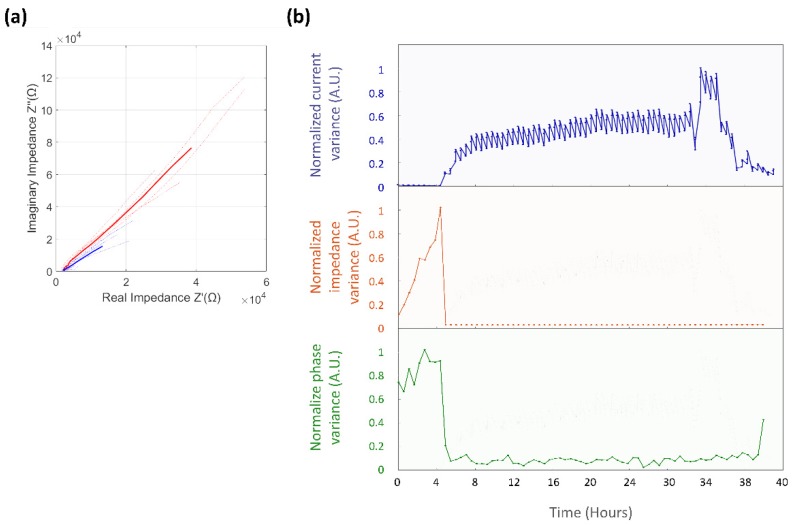
Analysis of EIS impedance and phase variance for microsystem colonization event experiment. (**a**) Nyquist plot for the EIS response before (Red) and after (Blue) the colonization event. (**b**) Comparison of the normalized variance for the CV current (Blue), EIS impedance (Orange) and EIS phase (Green).

The microsystem response for EIS measurements was recorded for the control experiment (Figure 12a) and for the previously observed colonization event (Figure 13a). The change ratio for the normalized impedance variance was 1.03, indicating a slight increase of the microsystem impedance over time, and of 0.89 for the normalized phase variance, as described in Figure 12b. The normalized variance of the impedance and phase response across EIS measurements was compared against the calculated CV current variance (Figure 13b). For the colonization event, a sudden drop in the values of impedance, with a change ratio of 0.15 ± 0.46, and phase variance, with a change ratio of 0.78 ± 0.45, were determined; this change was consistent in occurrence time with the time previously registered for the CV normalized current variance. In the case of the impedance variance, the value measured after the colonization event remained stable throughout the entire duration of the experiment, while the phase variance presented an increase in value after the electrode damage stage was reached.

The maximum frequency change for EIS measurements for both impedance and phase values was noticed at a mean frequency of 17.3 KHz, after where measurement became unstable.

**Figure A4:**
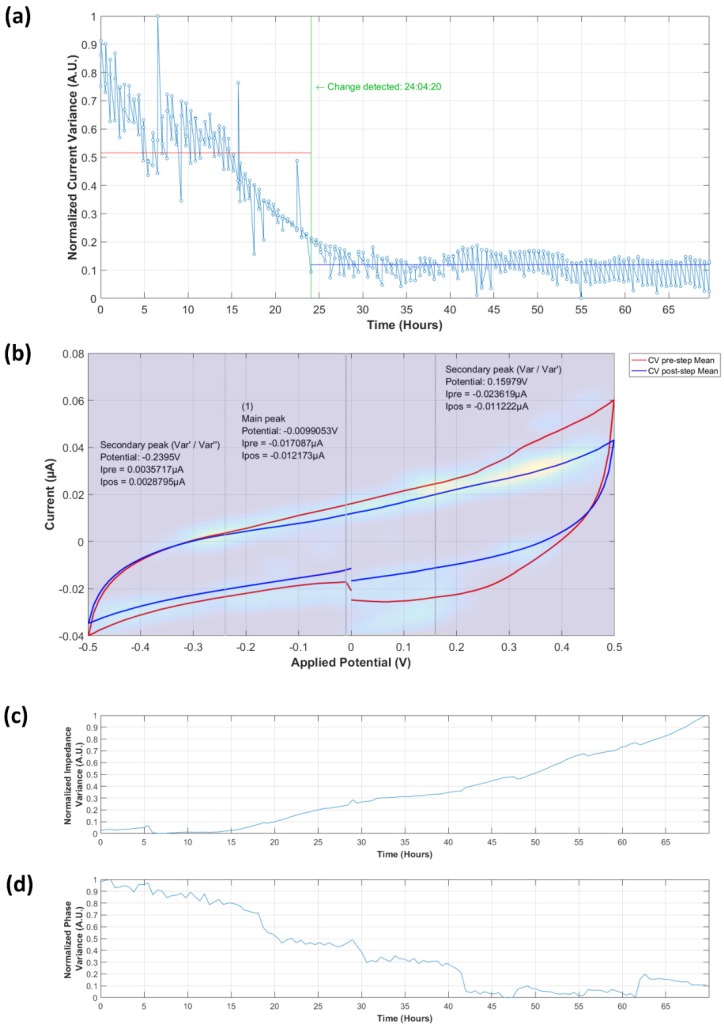
Recorded electrochemical response of a non-colonized microsystem for a ~70 h experiment with no inoculum. (**a**) Normalized current variance vs. time. Detected current peaks are indicated (peaks from variance to its first derivative ratio are indicated as Var/Var′, peaks from first derivative to second derivative ratio are indicated as Var′/Var″. The current values at each peak are indicated for the mean before colonization event as I_pre_ and for the mean after colonization event as I_pos_). Note that the software detected a change, but it does not have the characteristical step shape. (**b**) Recorded CV with calculated peaks and heatmap of detected peaks, where yellow represents a high concentration, and dark blue no peak. (**c**) Normalized impedance variance vs. time. (**d**) Normalized phase variance vs. time.

**Figure A5:**
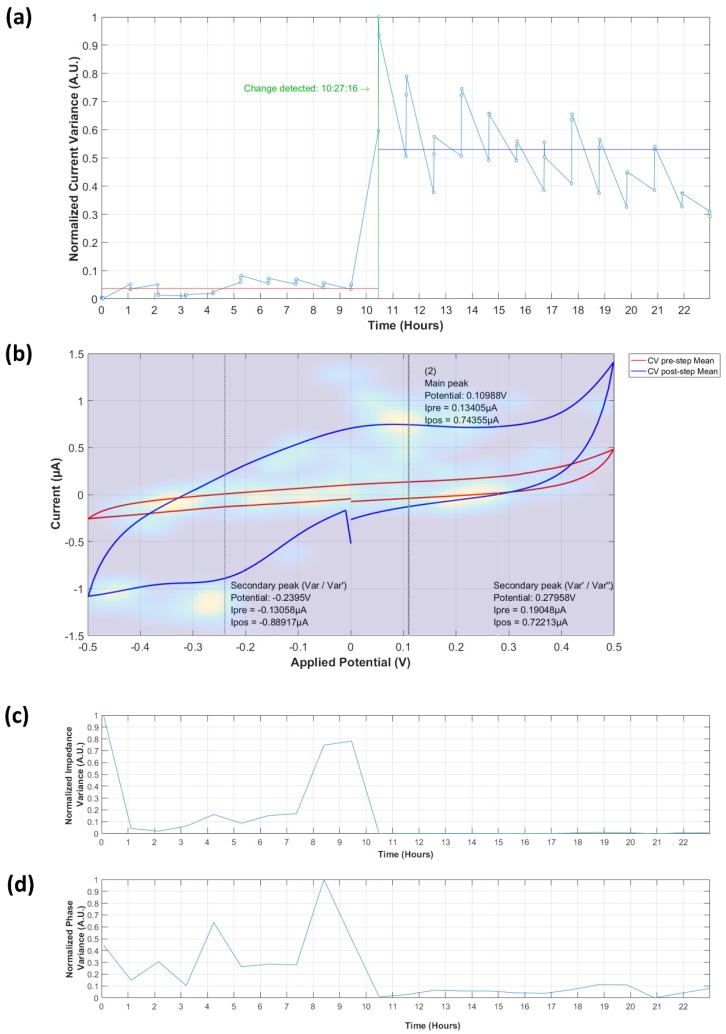
Recorded electrochemical response of a colonized microsystem for a ~24 h experiment. (**a**) Normalized current variance vs. time. Detected current peaks are indicated (peaks from variance to its first derivative ratio are indicated as Var/Var′, peaks from first derivative to second derivative ratio are indicated as Var′/Var″. The current values at each peak are indicated for the mean before colonization event as I_pre_ and for the mean after colonization event as I_pos_). Note the characteristical step shape. (**b**) Recorded CV with calculated peaks and heatmap of detected peaks, where yellow represents a high concentration, and dark blue no peak. (**c**) Normalized impedance variance vs. time. (**d**) Normalized phase variance vs. time.

**Figure A6:**
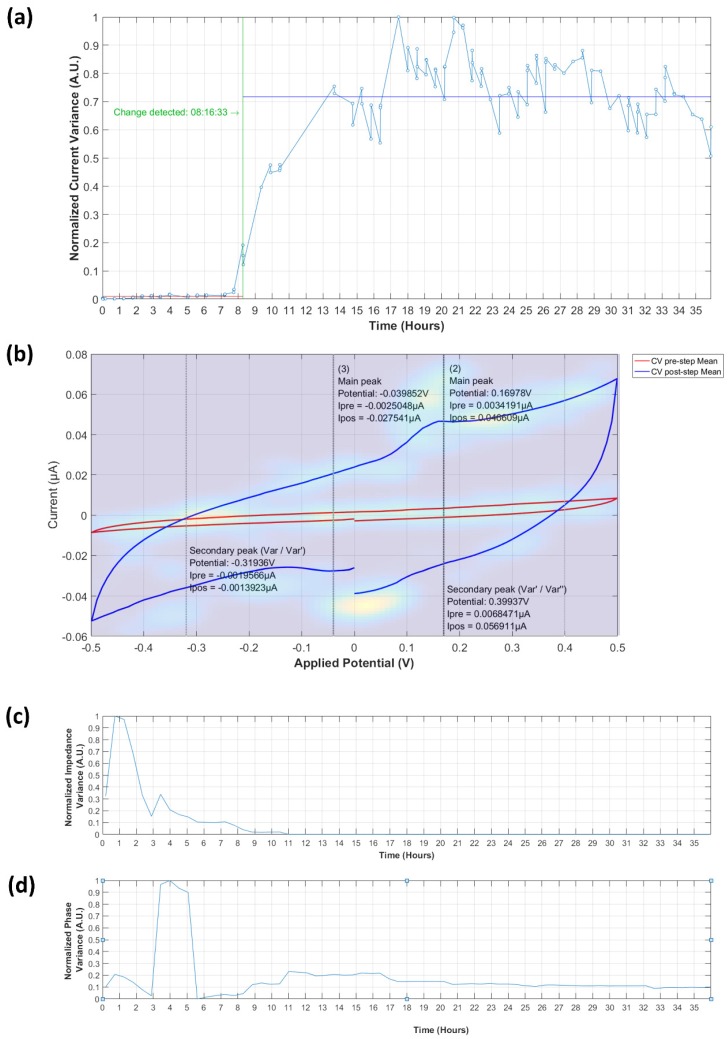
Recorded electrochemical response of a colonized microsystem for a ~36 h experiment. (**a**) Normalized current variance vs. time. Detected current peaks are indicated (peaks from variance to its first derivative ratio are indicated as Var/Var′, peaks from first derivative to second derivative ratio are indicated as Var′/Var″. The current values at each peak are indicated for the mean before colonization event as I_pre_ and for the mean after colonization event as I_pos_). Note the characteristical step shape. (**b**) Recorded CV with calculated peaks and heatmap of detected peaks, where yellow represents a high concentration, and dark blue no peak. (**c**) Normalized impedance variance vs. time. (**d**) Normalized phase variance vs. time.

**Figure A8:**
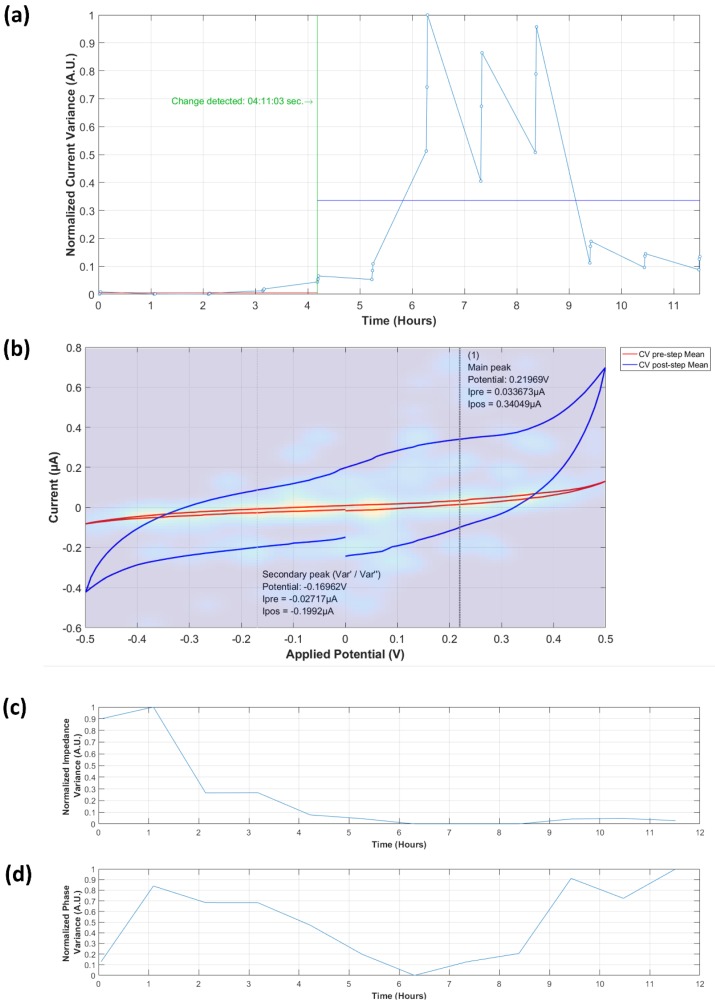
Recorded electrochemical response of a colonized microsystem and electrode damage. (**a**) Normalized current variance vs. time. Detected current peaks are indicated (peaks from variance to its first derivative ratio are indicated as Var/Var′, peaks from first derivative to second derivative ratio are indicated as Var′/Var″. The current values at each peak are indicated for the mean before colonization event as I_pre_ and for the mean after colonization event as I_pos_). (**b**) Recorded CV with calculated peaks and heatmap of detected peaks, where yellow represents a high concentration, and dark blue no peak. (**c**) Normalized impedance variance vs. time. (**d**) Normalized phase variance vs. time. Note that after the initial step-shaped curve, a sudden drop occurred in the current normalized variance, while no immediate change was seen in the impedance normalized variance, and an increase in the phase normalized variance occurred. These effects might be explained due to an increase in the microsystem contact resistance, while the faradaic effects in the microsystem are still present.

When no growth was observed, the CV normalized current variance, normalized impedance variance and phase variance figures did not show the described pattern in Figure 10. For the control experiments without inoculum (Figure A4), normalized variances showed specific trends without sudden changes. These sudden changes in all three normalized variances were only observed when a colonization event took place (Figure 10 and Figure A4). Figure A5 shows a colonization event, showing the sudden changes in all normalized variances, but after ~8 h, the microelectrodes presented physical damage that could be observed by the decrement of the normalized current variance and increment of the normalized phase variance. These effects might be explained due to an increase in the microsystem contact resistance, while the faradaic effects in the microsystem were still present. Figure A6 presents the early damage of the microelectrodes without bacterial colonization. As shown, only two of the three normalized variances presented sudden changes, attributed to an increase in the overall microsystem resistance, but with no faradaic effect. Damage of the microelectrodes was observed after multiple uses and were attributed to wear-off effects.

#### 3.4.2. Equivalent Circuit Model Fitting

**Figure 14:**
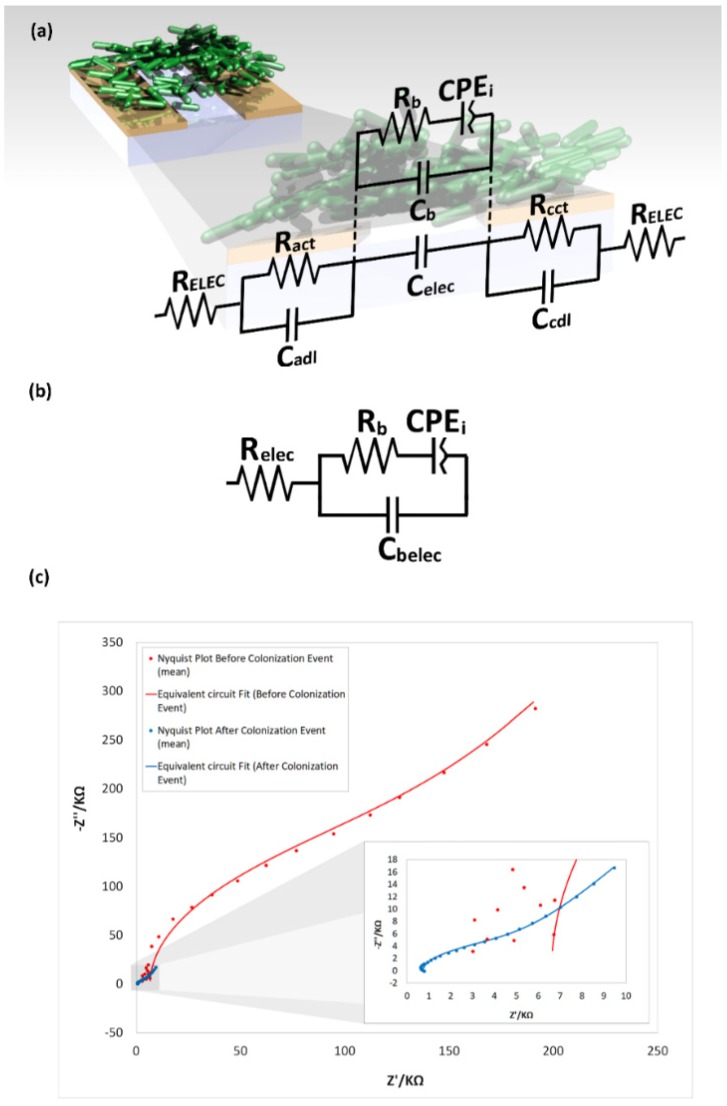
Equivalent circuit model for EIS analysis. (**a**) Equivalent circuit model for the generalized case. (**b**) Simplified equivalent circuit. (**c**) Nyquist plot for the collected (dots) and the fitted (continuous line) data of the microsystem before (red) and after (blue) the colonization event.

The rigorous equivalent circuit model for the microsystem generalized case is presented in Figure 14a. This model integrates two classical Randless cells (*R_ELEC_* + (*R_ct_||C_dl_*)) to describe the influence of the microsystem resistance (*R_ELEC_*) in series with the admittance of the electrochemical interface at the microelectrodes, anode and cathode, controlled by a charge transfer resistance (*R_act_* and *R_cct_*) and a double layer capacitance (*C_adl_* and *C_cdl_*). The capacitive effect, induced by the proximity of the two microelectrodes, is symbolized as a capacitive bridge between the two equivalent Randless cells (*C_elec_*). The bacterial cells were modeled after a modified Randless cell ((*R_b_ + CEP_i_*)*||C_b_*) [68,69,70]. The bacterial capacitance is indicated by a single capacitor (*C_b_*) in parallel with the charge transfer resistance of the bacteria (*R_b_*) and a constant phase element (*CPE_i_*) that represents the non-uniform accumulation of adsorbed species at the microelectrode surface.

This equivalent circuit model for the microsystem generalized case can be described as a solid cell with a blocking electrode in which the dielectric layer is modified by the bacterial presence. The complete equivalent circuit can be simplified, as shown in Figure 13b. The microelectrode double layer capacitance at the electrochemical interface can be neglected; this is primarily due to the effect of the reduced area of the microelectrode, which characteristically directly influences the represented electrochemical system time constant τ, determined by the resistor-capacitor product (RC) of the electrical components in this part of the circuit. As the dimensions become smaller, the electrode capacitance, which is proportional to the electrode area, decreases, and so does τ. The contribution of these elements is the only noticeable at frequencies above 10 MHz, which is two orders of magnitude larger than the maximum frequency used in this study. The microsystem overall resistance can also be simplified and represented with a single resistance (*R_elec_* = *R_ELEC_ + R_ELEC_*), as well as the microelectrode–bacteria interface capacitance which accounts for the of the capacitive induced effect in the microelectrodes and the surface interaction with the colonizing bacteria and can be expressed as the combination of the two parallel capacitances (*C_belec_* = *C_elec_*||*C_b_*).

The simplified equivalent circuit model was used to fit the data of the microsystem both before and after the bacterial colonization event (Figure 13c) with an average error for the calculated elements of the simplified equivalent circuit below 5% (excluding *R_ELEC_*, with an average error before the colonization event above 40%, and after the colonization event below 14%). The average values for the individual elements of the simplified equivalent circuit of the microsystem before and after the colonization event are summarized in Table 1.

The values of the individual elements were found to display important magnitude variations around the moment of the colonization event. The initially high resistance of the charge transfer resistance of the bacteria *R_b_*, with a calculated mean of 5.7 MΩ, had the most prominent change among all the electrical components of the equivalent circuit, with an average 48-fold decrease after the bacterial colonization event; this change was not surprising, as in the absence of bacteria, no faradaic process can be developed and thus the behavior of the charge transfer resistance is equivalent to an open circuit. The microsystem overall resistance, *R_elec_*, with an initial average value of 16 KΩ, also displayed a magnitude decrease; however, these values were associated to a different phenomenon: the presence of stray potentials. Before the bacterial colonization event, *R_elec_* presented with very unstable behavior. The early arrival of bacteria to the microelectrode vicinity was accompanied by an initial decrease of the resistance value and the stabilization of the measured resistance values. As the bacteria arrives to the microelectrode surface, an electrical contact is generated, thus further stabilizing the measured value of the resistive element.

**Figure 15:**
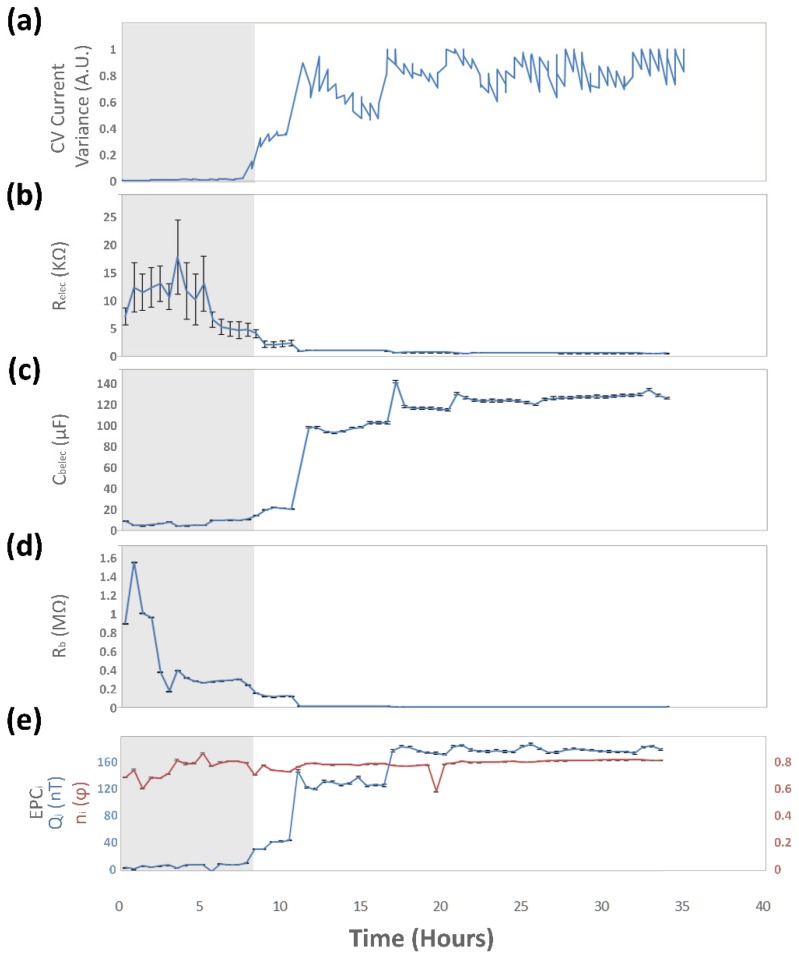
Changes of the simplified equivalent circuit over a 35-h period with a bacterial colonization event. (**a**) CV current variance analysis of the microsystem. (**b**) Microsystem resistance. (**c**) Electrode–bacteria interface capacitance. (**d**) Charge transfer resistance of the bacteria. (**e**) Constant phase element for the non-uniform accumulation of species at the electrode surface.

Once the bacteria are in contact with the microelectrode surface, the possible faradic processes can occur. This is confirmed by the five-fold increase of the bacterial–electrode interface double layer capacitance, *C_belec_*, with an initial mean value of 920 pF. The increase on the constant phase element magnitude, *CPE_i_*, with a mean value of 31.4 nT 0.8 φ before the colonization event, and a 1.5-fold increase in magnitude (Q), is a particularity of this microsystem. In traditional bacterial electrochemical systems, the value of the constant phase element for adsorbed species is expected to decrease over time. This behavior is explained as the aggregation of cells to an electrode surface in a liquid medium would act as a capacitive coating, interfering with the active electron exchange between the electrodes and the medium [71]. In the presented microsystem, the opposite effect takes place: prior to the bacterial colonization event, no electron exchange can be detected, which correlates to the fact that no active species are present at the electrode–gas interface of the empty microsystem. The evolution of the values over time of the simplified circuit elements for a colonization event, as registered by the CV, detected after 8 hours, for a 35-hour experiment, are shown in Figure 15.

## 4. Discussion

The development of a faradaic contribution to the microsystem was evidenced for the CV response and discussed in Section 3.3.2; this contribution possibly corresponds to the redox reaction attributed to the transfer of electrons at the bacteria–microelectrode interface [36,55,56,72,73]. This assumption should be carefully addressed, as such mechanisms are still poorly understood [74], and require a more comprehensive examination. Further work should be conducted on this matter to better understand the mechanisms involved and the influence in the observed redox reaction. Additionally, extensive research on the nature of the EET process in bacteria will help to understand the elements that take part in this process and their role in energetic systems for bacterial metabolism. The presented microsystem can potentially provide a powerful tool to evaluate such mechanisms; in combination with genetic manipulation and analysis tools, high resolution visualization, and other techniques, new insights can be achieved related to the EET process and its components.

The microsystem sustained up to 72 h of continuous use with no sign of deterioration of the polymeric microstructure, which makes it a good candidate for long-term live-cell imaging and characterization, with the potential to be considered as a reusable device. Additionally, modification of the structure can be explored; in this way, the influence of the microsystem geometry, the interaction with other species, the chemical response and the environmental influence on behavior can be studied.

The reliability of the microsystem was considered low; however, it was noticed that despite that the continuous CV measurement resulting in a gradual increase of the current magnitude measured at the microelectrodes, the microsystem rapidly recovered to the initial condition after a brief pause. In addition, microelectrode damage was also observed and characterized, although these damages were observed after several hours of use and interaction with bacteria colonization events. Therefore, even if there are physical changes in the environment, such as pH changes (not studied in this work) due to bacterial presence, the microelectrodes lasted long enough to be able to study the phenomena.

Future work should be directed to identify the limitations of the current project in regard to the discrimination on the expression of the reactions that occur on the bacterial membrane and the ones that take place on the extracellular matrix of the bacterial community. Increasing the stability of the electrodes and the possibility to implement surface modifications to enhance the electrochemical response would greatly widen the possibilities of usage of this microsystem and could help to disseminate its use and penetration on the scientific community.

This microsystem allows an encouraging range of applications in the scope of bacterial physiological characterization and electrochemical evaluation that can be integrated in real-life applications for bacterial development quantification, biosensing, and drug-test evaluation. We are confident that it can be integrated to routine characterization of bacterial BES and would significantly contribute in the exploration of the mechanisms of EET.

The presented microsystem exhibited good performance regarding propagation, control of cell culture and a readily available electrochemical response. Moreover, it would be possible to develop a mathematical model to describe the colonization along the microsystem; this can be advantageous to quantitatively characterize the behavior of a supported bacterial strain and offer an alternative method to calculate growth rates, identify infection treatments and control proliferation. Further studies should be conducted to characterize cell viability within the microsystem. An optical, real-time method would be advantageous and easily integrated with the presented microsystem.

## 5. Conclusions

In this paper we presented a fully automated platform that allows direct visualization of bacteria in a non-fluidic microsystem and the unmediated interaction with gold microelectrodes to enable electrochemical characterization. The microsystem relies on the aggregation of bacteria in a confined environment to force it in close contact with the microelectrodes, with a potential application for the study of the electron transfer mechanisms of bacteria and their surrounding environment.

The growth monitoring and characterization of *E. coli*, presented in Section 3.2, was used to allow an initial approach to the growth dynamics inside the microsystem. This monitoring eased the growth assessment of the bacterial culture and set the foundation to evaluate the reliability of the electrochemical procedures. The electrochemical characterization of the normalized variance for the CV and EIS measurements, presented in Section 3.3.1 and Section 3.4.1, was used to allow the evaluation of the electrochemical measurements. A large amount of data was extracted from the experiments, and rapid evaluation using the developed computer algorithm of the microsystem was beneficial for this study. Furthermore, this method allows the observation of small changes in a graphic fashion and provided a tool to quantitatively determine the overall response of the microsystem. The application of this method confirmed a direct correlation with the variance of the electrochemical response and the observed colonization events.

The increase in the current response of the non-faradaic component of the CV measurement and the decrease of the overall impedance measured at the EIS response were both related to the formation of a conductive surface between the electrodes as the bacteria aggregated in the three-dimensional confined space.

The equivalent circuit model based on the EIS measurements, presented in Section 3.4.2, provides complementary evidence of the existence of a faradaic process at the bacteria–electrode interface and can be potentially used to describe changes due to external factors, allowing us to use the presented microsystem as a biosensor platform.

## Figures and Tables

**Table 1:** Average values of the simplified equivalent circuit.

Electric Component		Value before Colonization Event	Value after Colonization Event	Unit
*C_belec_*		0.919	6.72	nF
*R_elec_*		15.96	3.904	KΩ
*R_b_*		5.71	0.107	MΩ
*CPE_i_*	*Q_i_*	31.37848 × 10^−9^	69.78041 × 10^−9^	T
	*n_i_*	0.77428	0.865045	φ

*C_belec_*: bacterial–electrode interface double layer capacitance; *R_elec_*: microsystem overall resistance; *R_b_*: charge transfer resistance of the bacteria; *CPE_i_*: Constant phase element $Z\left(\omega \right)=\frac{1}{Q_{i}{\left(j\times \omega \right)}^{n_{i}}}$; *Q_i_*: $\left[T\right]=S\times s^{n_{i}}$; *n_i_*: $\left[\varphi \right]=90^{{}^{\circ}}\times n_{i}$.

**Figure A3:**
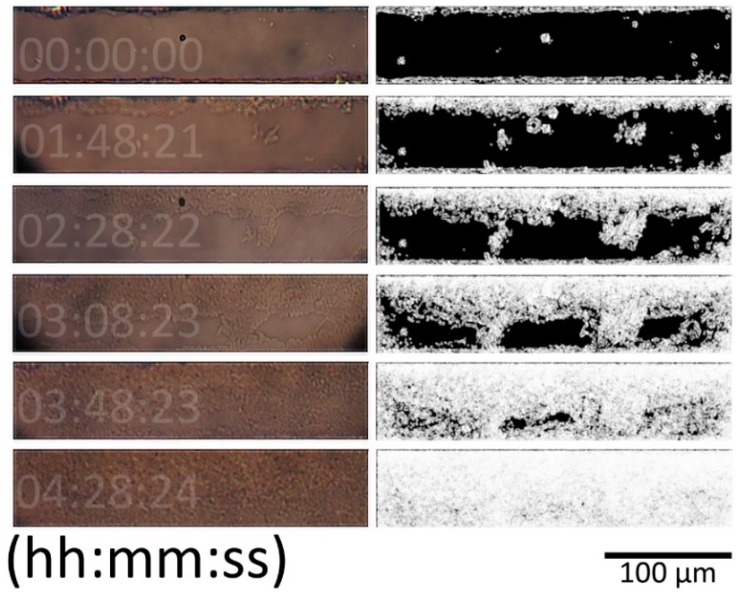
Microchannel section micrographs and processed images for bacterial growth estimation.

**Figure A7:**
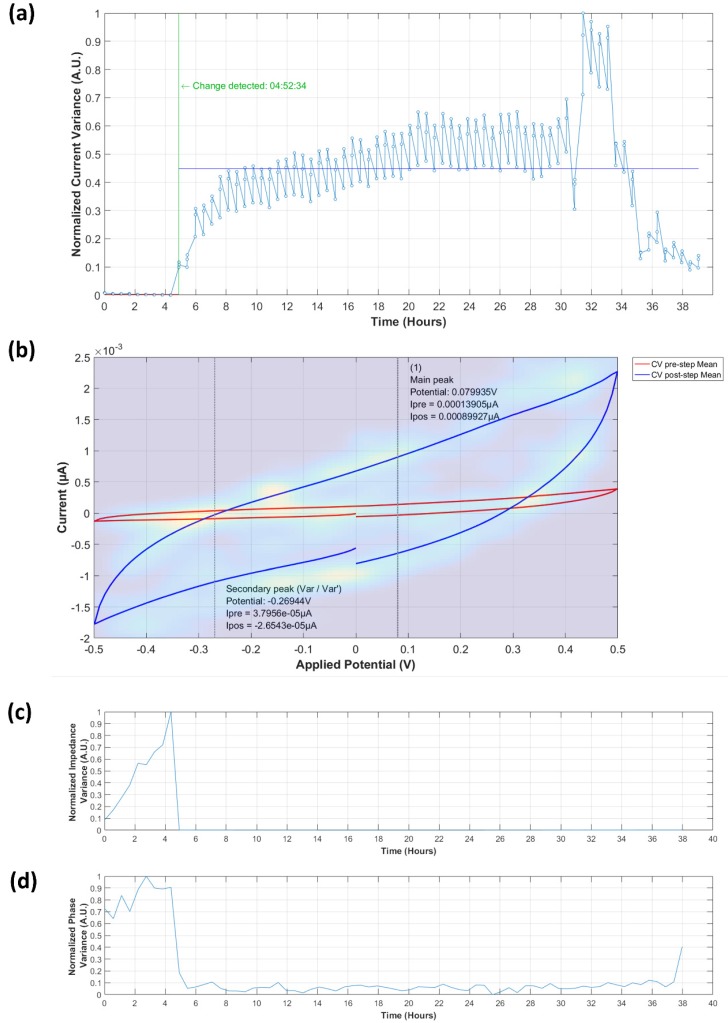
Recorded electrochemical response of a colonized microsystem for a ~38 h experiment. This experiment had a lower reported current density, and damage of the electrode can be observed at ~31 h. (**a**) Normalized current variance vs. time. Detected current peaks are indicated (peaks from variance to its first derivative ratio are indicated as Var/Var′, peaks from first derivative to second derivative ratio are indicated as Var′/Var″. The current values at each peak are indicated for the mean before colonization event as I_pre_ and for the mean after colonization event as I_pos_). Note the characteristical step shape. (**b**) Recorded CV with calculated peaks and heatmap of detected peaks, where yellow represents a high concentration, and dark blue no peak. Note that the anodic peak is low; this can be a result of high resistance due to the electrode wearing off as a result of extended use. (**c**) Normalized impedance variance vs. time. (**d**) Normalized phase variance vs. time.

**Figure A9:**
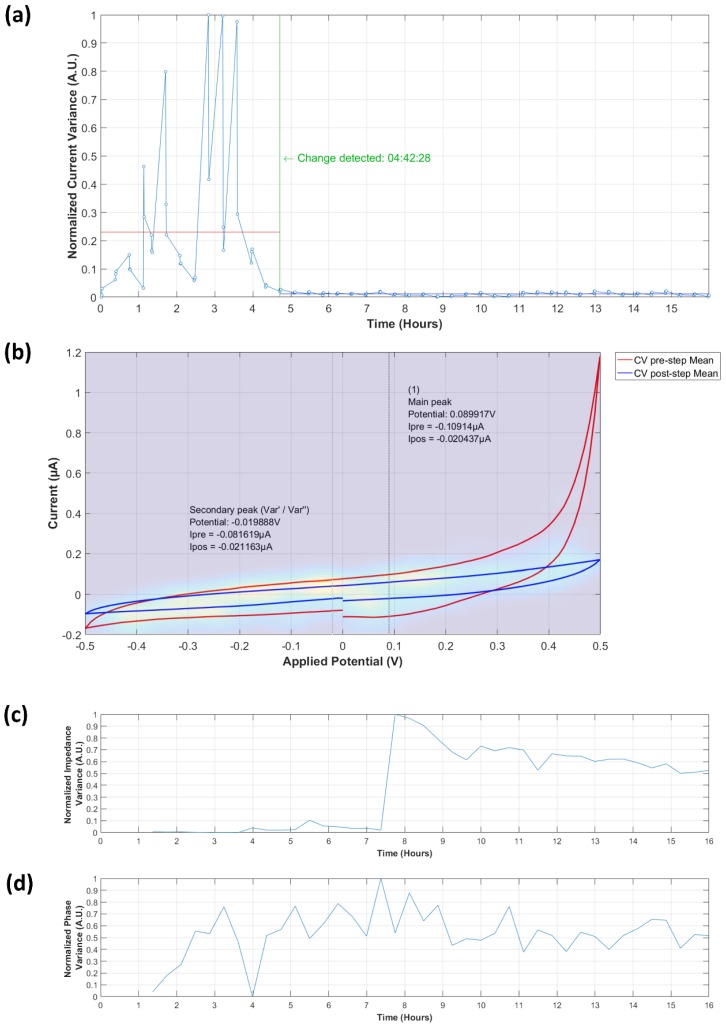
Recorded electrochemical response of early electrode damage without a colonization event. (**a**) Normalized current variance vs. time. Detected current peaks are indicated (peaks from variance to its first derivative ratio are indicated as Var/Var′, peaks from first derivative to second derivative ratio are indicated as Var′/Var″. The current values at each peak are indicated for the mean before colonization event as I_pre_ and for the mean after colonization event as I_pos_). (**b**) Recorded CV with calculated peaks and heatmap of detected peaks, where yellow represents a high concentration, and dark blue no peak. (**c**) Normalized impedance variance vs. time. (**d**) Normalized phase variance vs. Time. This incident was registered mainly due to wear-off of the electrodes after repeated use. An increase of the current normalized variance and the impedance normalized variance, with no indicative change in the phase normalized variance, were attributed to an increase in the overall microsystem resistance while no faradaic effect was present.
